# The association of osteoporosis and geriatric syndromes in the elderly: data from the Russian epidemiological study EVKALIPT

**DOI:** 10.1007/s11657-023-01217-x

**Published:** 2023-02-13

**Authors:** Ekaterina N. Dudinskaya, Natalia M. Vorobyeva, Julia S. Onuchina, Lubov V. Machekhina, Elena V. Selezneva, Lilia N. Ovcharova, Yulia V. Kotovskaya, Olga N. Tkacheva

**Affiliations:** 1 Age-Related Endocrine and Metabolic Disorders Laboratory, Russian Gerontology Research and Clinical Centre, Pirogov Russian National Research Medical University, Moscow, Russia; 2 Laboratory of Cardiovascular Aging, Russian Gerontology Research and Clinical Centre, Pirogov Russian National Research Medical University, Moscow, Russia; 3 Department of Aging Diseases, Faculty of Additional Professional Education, Russian Gerontology Research and Clinical Centre, Pirogov Russian National Research Medical University, Moscow, Russia; 4 Institute for Social Policy, National Research University “Higher School of Economics”, Moscow, Russia; 5 Russian Gerontology Research and Clinical Centre, Pirogov Russian National Research Medical University, Moscow, Russia; 6 Russian Academy of Sciences, Moscow, Russia

**Keywords:** Older age, Osteoporosis, Fractures, Geriatric syndromes

## Abstract

***Summary
*:**

Osteoporosis is associated with almost all geriatric syndromes (GSs), and the occurrence of osteoporosis in patients over 65 years of age increases by 1.2–2.5 times. Early diagnosis of osteoporosis and GSs is very important. Additional programs should be adopted by the state to introduce information about the possibilities of working with elderly patients.

**Purpose:**

To analyze associations of osteoporosis with geriatric syndromes in patients aged 65 years and older in the Russian Federation.

**Methods:**

A total of 4308 patients (30% men) aged 65–107 years were examined and distributed into 3 age groups (65–74 years, 75–84 years, and 85 years and older). All patients underwent a comprehensive geriatric assessment. In the “Falls and risk of falls” module, the number and circumstances of falls over the previous year were analyzed, as well as the history of fractures. The presence of osteoporosis was determined based on medical records. Physical examination included anthropometric measurements and standard enquiry, short physical performance battery (SPPB), dynamometry, measurement of gait velocity, Mini-Cog test, and orthostatic test.

**Results:**

A total of 507 patients (11.8%) had evidence of osteoporosis; indications of low-energy fractures in history were recorded in 739 (17.3%) patients. Patients with osteoporosis were older, shorter, and predominantly women; had a lower body weight and a higher Charlson comorbidity index; and took more drugs. Patients with osteoporosis had lower gait velocity, hand grip strength, Barthel index value, and scores of the Lawton instrumental activities of daily living scale, the MNA (Mini Nutritional Assessment) short-form, and the SPPB. Osteoporosis is associated with almost all geriatric syndromes (GSs), and the occurrence of osteoporosis in patients over 65 years of age increases by 1.2–2.5 times.

**Conclusions:**

Osteoporosis is associated with almost all GSs. The association of osteoporosis with advanced GSs aggravates the condition of these patients. Early diagnosis of osteoporosis and GSs is very important. Additional programs should be adopted by the state to introduce information about the possibilities of working with elderly patients: early detection and correction of osteoporosis.

## Introduction

The global population is aging, and the proportion of the elderly people is registered in on the rise worldwide. In the middle of the twentieth century, the proportion of people over 65 years of age was 7.7% of the world’s population; over the first 20 years of the next century, the proportion of people of this age increased to 19% (2019 data), and by the middle of the twenty-first century (2050), it will be at least 27% [1]. As the population rapidly ages, interest in age-related diseases and geriatric syndromes (GSs) also rises. GS is a multifactorial age-associated clinical condition that worsens the quality of life and increases the risk of adverse outcomes (death, dependence on external assistance, repeated hospitalizations, and need for long-term care) and functional disorders [2, 3]. Age-associated diseases and GSs include disease such as osteoporosis. The term “osteoporosis” was used in France in the early nineteenth century and implied bone pathology. Osteoporosis is classified by WHO as one of the five most significant human diseases alongside infarction, stroke, cancer, and sudden death. In the mid-2000s, 33.8% of women and 26.9% of men in Russia over the age of 50 have osteoporosis. Extrapolation of the entire population of the Russian Federation revealed 14 million patients, which is about 10% of the population [4]. At present, the social significance of osteoporosis is recorded, determined by its consequences, namely low-trauma fractures of the vertebral bodies and bones of the peripheral skeleton, leading to high healthcare burden as well as significant impairments such as disability and mortality [5, 6].

At the same time, the exact prevalence of osteoporosis is unknown due to the fact that in most cases, only fractures of the proximal femur are recorded, including in elderly patients.

In Russia, data on osteoporosis is old, and generally relates to particular regions, that is why it needs to provide new investigation.

In 2018, the EVKALIPT (Epidemiological study of the prevalence of GSs and age-associated diseases in the elderly patients in regions of the Russian Federation with different climatic, economic, and demographic characteristics) study among patients aged 65 years and older was started, aimed at obtaining the prevalence of age-associated diseases, frailty syndrome, other GSs, osteoporosis, and fractures, as well as analysis of their contribution to parameters of general health and functional status in Russia. The study was performed from 2018 to 2020 in 11 regions of Russia at the initiative of the Russian Association of Gerontologists and Geriatricians and the Russian Gerontological Research and Clinical Center in cooperation with the National Research University Higher School of Economics.

## Materials and methods

In a cross-sectional analytical epidemiological study, EVKALIPT included patients aged from 65 to 107 years (mean age 78 ± 8 years), from April 2018 to October 2019, who live in 11 Russian regions (Republics of Bashkortostan, Dagestan, and Chuvashia; Voronezh and the Voronezh region; Moscow; Saratov; St. Petersburg and the Leningrad region; Ivanovo, Ryazan, Samara, and Smolensk region). Inclusion criteria were age of 65 years or older and written voluntary informed consent to participate in the study. In accordance with the protocol, participants were distributed into three age groups (65–74 years, 75–84 years, and ≥ 85 years).

Initially, it was planned to include 600 people from each region (200 participants in each age group) in the study. However, only a small number of participants were included in this way. Most of the participants were recruited on the basis of seeking medical care, i.e., planned inpatient treatment in geriatric departments/hospitals, so the actual total number of participants and their distribution by age groups in individual regions did not always correspond to the planned. The majority (60%) of the participants were examined in a clinic, every fifth in a hospital (20%) or at home (19%), and 1% in nursing homes.

This data is a part of a large epidemiological study of EVKALIPT conducted in the Russian Federation. Russian EVKALIPT study protocol and basic characteristics of participants are summarized in the article [7].

All patients underwent a comprehensive geriatric assessment (CGA) in two stages: (1) survey based on a specially designed questionnaire and (2) physical examination. This was performed simultaneously by a geriatrician and a geriatric nurse at the patient’s residence (in a hospital, clinic, residential institution/assisted-living facility, or at home).

The questionnaire included the “Socio-economic status,” “Occupational history,” “Risk factors for chronic non-communicable diseases,” “Chronic non-communicable diseases,” “Drug therapy,” “Obstetrics and gynecological history,” “Falls and risk of falls,” “Chronic pain,” “Sensory deficits,” “Oral health,” “Urine and fecal incontinence,” “Use of aids,” “Laboratory examination results,” and a number of standardized scales: screening scale “Age is not a problem,” the Geriatric Depression Scale GDS-15, Basic Functional Activity Scale (Barthel Index), Lawton Instrumental Activities of Daily Living Scale, MNA (Mini Nutritional Assessment) short-form, Charlson comorbidity index, and Visual Analog Scale (VAS) for self-assessment of quality of life, health status, and intensity of pain syndrome at the time of the examination and during their stay.

For screening frailty in daily practice in Russia, they developed the questionnaire “Vozrast ne pomekha” (VNP), which translates into English as “Age is not a hindrance.” The study questionnaire was composed of seven dichotomous items for evaluation of the following characteristics: weight loss (“Did you lose 5 kg or more in the past 6 months?”), impaired vision or hearing (“Do you have any restrictions in daily living due to decreased vision or hearing?”), fall-related injuries (“Have you had any injury-related falls during the last year?”), mood disorder (“Have you felt depressed, sad or anxious over the past weeks?”), cognitive impairment (“Do you have problems with memory, comprehension, orientation or ability to plan?”), urinary incontinence (“Do you have urinary incontinence?”), and difficulty walking (“Do you have any difficulty walking at home or on the street up to a distance of 100 m, or climbing a flight of stairs?”). One point was recorded for each positive answer, so the total score ranged from 0 to 7 [8]. A score of ≥ 5 indicates a high probability of frailty syndrome. This questionnaire underwent a validation process, and the results of that process were published previously and are presented in abridged form here [9]. This scale was added in clinical guidelines on frailty in Russia [10].

The physical examination included (1) short physical performance battery tests (SPPB), (2) dynamometry, (3) measurement of gait velocity, (4) Mini-Cog test, (5) measurement of height and body weight, calculation of body mass index (BMI), (6) measurement of blood pressure (BP) and heart rate (HR), and (7) orthostatic test.

All tests, scales, and questionnaires used in the study (with the exception of the Charlson comorbidity index) are in the Russian clinical guidelines “Frailty syndrome” [10, 11]. The detailed study protocol and baseline characteristics of the participants have been presented in our previously published article [7].

The “Falls and risk of falls” module considered the number and circumstances of falls over the previous year, as well as the history of fractures (fractures of the vertebrae, femur, and radius when falling from standing height and their number, surgical treatment for fractures of the vertebrae, the need for care due to fracture, fracture of the femoral neck in parents).

The physician assessed cognitive function and completed the modules “Chronic non-communicable diseases,” “Drug therapy,” “Obstetrics and gynecological history,” and “Laboratory examination results.” The nurse completed all other modules and the physical examination.

The presence of the following GSs was determined: (1) frailty syndrome, (2) cognitive impairment, (3) depression, (4) malnutrition, (5) orthostatic hypotension, (6) urinary incontinence, (7) fecal incontinence, (8) functional disorders, (9) loss of autonomy, (10) falls (for the previous year), (11) vision deficit, (12) hearing loss, (13) sensory deficit (any), (14) chronic pain syndrome, and (15) bedsores.

Osteoporosis was diagnosed if in medical history is registered a low-trauma (i.e., fragility) fracture of major bones (vertebral or proximal femur fracture or multiple fracture), increased fracture risk using FRAX® (Fracture Risk Assessment Tool) Russian-specific threshold, or if patient had results of Bone Densitometry with *T*-score − 2.5 or below in the lumbar spine, femoral neck, total proximal femur, or patient’s information. A fragility fracture was mean as a fracture sustained from low-energy trauma, such as a fall from standing height or less, that would not have occurred in healthy bone, excepting fractures of the skull, face, fingers, and toes.

### Characteristics of the participants

The study included 4308 patients (30% men) aged 65–107 years (Table 1). The majority (60%) of participants were examined in a polyclinic setting, 20% examined in a hospital, 19% at home, and 1% in residential institutions/assisted-living facilities. Among those examined, overweight patients prevailed (41%), while the proportion of patients with obesity and normal body weight was similar (30% and 28%), and 1.3% of participants were underweight (Table 1). Among patients with obesity, the majority of participants were obesity type I. With an increase in age, there is a decrease in height, body weight, BMI, the proportion of obese patients, and the severity of obesity, as well as an increase in the proportion of patients with normal weight. The proportion of overweight patients was identical in all age groups. The mean values of systolic and diastolic BP and heart rate were within the normal range in all patients; however, diastolic BP also decreased with age and pulse BP increased with similar identical values of systolic BP and HR.

**Table 1:** Demographic, anthropometric, and clinical characteristics of patients aged 65 years and older (values in bold indicates statistical difference)

Parameter	All patients (*n* = 4308)	Age groups			*p* for trend
		65–74 years (*n* = 1583)	75–84 years (*n* = 1519)	≥ 85 years (*n* = 1206)	
Age, years (M ± SD)	78.3 ± 8.4	69.1 ± 2.6	79.4 ± 2.5	88.9 ± 3.3	-
Male gender, %	29.7	31.9	27.3	29.9	**0.020**
Height, m (M ± SD)	1.63 ± 0.09	1.64 ± 0.08	1.62 ± 0.08	1.61 ± 0.09	** < 0.001**
Weight, kg (M ± SD)	73.9 ± 14.3	78.3 ± 14.5	73.3 ± 13.3	68.9 ± 13.2	** < 0.001**
Body mass index, kg/m^2^ (M ± SD)	27.9 ± 5.0	29.0 ± 5.2	27.9 ± 4.9	26.6 ± 4.4	** < 0.001**
Body mass, %					
Deficit	1.3	1.0	0.9	2.2	**0.007**
Norm	27.6	21.3	28.4	34.7	** < 0.001**
Excess	40.9	41.1	39.6	42.2	0.414
Obesity	30.2	36.6	31.1	21.0	** < 0.001**
Degrees of obesity, % (*n* = 1264)					
I	72.2	66.8	75.0	78.8	**0.001**
II	21.6	24.2	20.2	18.4	0.118
III	6.3	9.0	4.8	2.8	**0.001**
Systolic blood pressure, mm Hg (M ± SD)	136.1 ± 16.5	136.4 ± 16.6	136.0 ± 16.0	135.8 ± 17.0	0.819
Diastolic blood pressure, mm Hg (M ± SD)	80.2 ± 9.5	81.6 ± 9.5	80.1 ± 9.2	78.5 ± 9.7	** < 0.001**
Pulse blood pressure, mm Hg (M ± SD)	55.9 ± 13.0	54.8 ± 12.5	55.8 ± 12.4	57.3 ± 14.0	** < 0.001**
Heart rate, beats/min (M ± SD)	72.7 ± 8.6	72.6 ± 8.3	73.0 ± 9.1	72.3 ± 8.3	0.111

### Statistical data analysis

The study was performed using IBM® SPSS® Statistics version 23.0 (SPSS Inc., USA). The type of distribution of quantitative variables was analyzed using the one-sample Kolmogorov–Smirnov test. Results of parametric data were presented as M ± SD, where M is the mean, and SD is the standard deviation; while that of non-parametric data, the results are presented as Me (25%; 75%), where Me is the median, and 25% and 75% are the 25th and 75th percentiles. For clarity, some variables are presented simultaneously as Me (25%; 75%) and M ± SD. For intergroup comparisons, the Mann–Whitney, Kruskal–Wallis, and Pearson’s *χ*^2^ tests, as well as Fisher’s two-tailed exact test, were used. Relationships between variables were assessed using binary logistic regression with the odds ratio (OR) and 95% confidence interval (CI). One-way and multivariate regression analyses were performed after adjusting for age and gender. We analyzed the variables using the direct stepwise selection method of multivariate analysis. Differences were considered significant at a two-tailed *p* < 0.05.

## Results

The presence of osteoporosis was determined based on past medical records. Information related to chronic disease was present for 4295 (99.7%) participants, and 507 (11.8%) were osteoporotic. Information about drugs was known in 501 (98.8%) patients with osteoporosis, and almost half (49.1%) of them were not on treatment of osteoporosis. Among all patients treated with drugs, the majority (91.4%) were on calcium and vitamin D supplements, 12 (4.7%) patients received antiresorptive therapy, 1 (0.4%) patient received bone anabolic therapy, and 9 (3.5%) patients received antiresorptive and bone-metabolic therapy.

Information related to the history of fragility fractures was available in 4275 (99.2%) participants. A history of fragility fractures was recorded in 739 (17.3%) patients, and 292 (39.6%) had a fracture of the proximal femur, 464 (63%) had a fracture of the radial bone, and 158 (21.5%) had a fracture of the vertebrae. We noted a decrease in the proportion of patients undergoing surgical treatment for vertebral fractures alongside (Table 2); there were no differences in other parameters across the age groups.

**Table 2:** Incidence of low-energy fractures in history in patients aged 65 years or older (values in bold indicates statistical difference)

Parameter	All patients (*n* = 4308)	Age groups			*p* for trend
		65–74 years (*n* = 1583)	75–84 years (*n* = 1519)	≥ 85 years (*n* = 1206)	
History of low-energy fractures, %	17.3	15.9	17.7	18.6	0.148
Proximal femur fractures due to a fall from standing height in history, %	6.8	6.0	6.5	8.2	0.057
Surgical treatment for proximal femur fracture in history, % (*n* = 290)	57.6	58.1	60.6	54.1	0.647
Fractures of the radial bone due to a fall from standing height in history, %	10.8	9.8	11.5	11.3	0.257
Number of fractures of the radial bone due to a fall from standing height in history, % (*n* = 432)					**0.022**
1	80.8	87.5	75.0	80.5	
2 or more	19.2	12.5	25.0	19.5	
History of vertebral fractures due to a fall from standinsg height, %	3.7	3.2	3.7	4.3	0.289
Number of vertebral fractures due to a fall from standing height in history, % (*n* = 72)					0.562
1	86.1	91.3	80.8	87.0	
2 or more	13.9	8.7	19.2	13.0	
Surgical treatment for a vertebral fracture in history, % (*n* = 139)	33.8	46.5	34.7	21.3	**0.040**
Need for care due to a fracture, % (*n* = 154)					0.227
No	23.4	33.3	21.8	15.7	
Yes, cared for by a nurse	12.3	8.3	10.9	17.6	
Yes, cared for by relatives	64.3	58.3	67.3	66.7	
Proximal fracture in parents, %	3.4	3.6	3.4	3.1	0.817

**Fig. 1:**
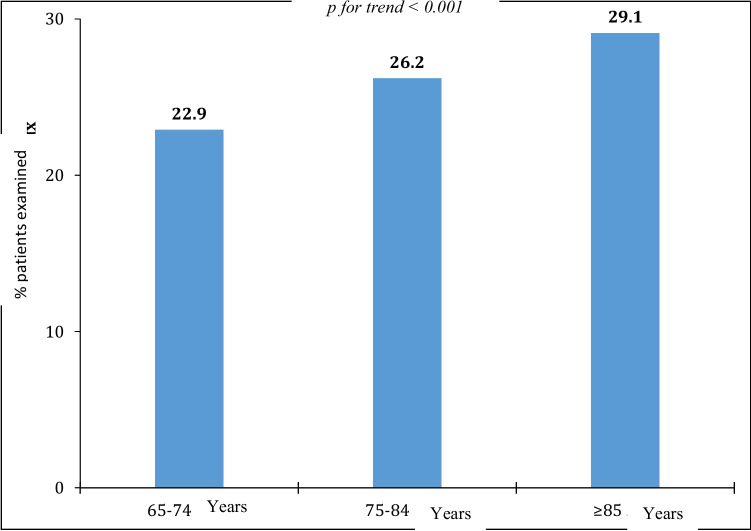
Prevalence of osteoporosis in patients aged 65 years and older according to age groups (patients with documented osteoporosis and with a history of fractures)

Among patients without documented osteoporosis (*n* = 3788), 594 (15.7%) presented with low-energy fractures in history: thus, these patients also had osteoporosis not diagnosed in a timely manner. For further analysis, patients with documented osteoporosis and with a history of fractures were combined into one group (group with osteoporosis; *n* = 1101). The osteoporosis group accounted for 25.6% of all patients. Comparison group consisted of patients without documented osteoporosis or history of low-energy fractures (group without osteoporosis; *n* = 3168). Increased prevalence of osteoporosis increased significantly with age (Fig. 1).

Patients with osteoporosis were older, shorter, and predominantly females; had a lower BMI and a higher Charlson comorbidity index; and took much more drugs (Table 3).

**Table 3:** Demographic, anthropometric, and clinical characteristics according to the presence or absence of osteoporosis in patients aged ≥ 65 years (*n* = 4269) (values in bold indicates statistical difference)

Indicator	Osteoporosis (*n* = 1101)	No osteoporosis (*n* = 3168)	*p*
Age, years (M ± SD)	79.2 ± 8.4	78.0 ± 8.4	** < 0.001**
Female gender, %	81.7	66.4	** < 0.001**
Height, m (M ± SD)	1.61 ± 0.08	1.63 ± 0.09	** < 0.001**
Weight, kg (M ± SD)	72.1 ± 15.2	74.5 ± 13.9	** < 0.001**
Body mass index, kg/m^2^ (M ± SD)	27.7 ± 5.2	28.0 ± 4.9	**0.026**
Body mass, %			
Deficit	1.6	1.2	0.374
Norm	29.1	27.0	0.192
Excess	41.4	40.7	0.689
Obesity	27.9	31.0	0.055
Degrees of obesity, % (*n* = 1256)			
I	68.5	73.3	0.105
II	24.2	20.9	0.229
III	7.4	5.8	0.337
Charlson comorbidity index [Me (25%; 75%)]	5 (4; 7)	4 (3; 6)	** < 0.001**
Charlson comorbidity index ≥ 5 points, %	60.0	49.0	** < 0.001**
Number of drugs	6 (4; 8)	5 (3; 7)	** < 0.001**
Number of drugs ≥ 5, %	68.2	56.0	** < 0.001**

According to the results of CGA, patients with osteoporosis had lower gait velocity, hand grip strength, Barthel index value, Lawton Instrumental Activities of Daily Living Scale scores, MNA (Mini Nutritional Assessment) short-form, and SPPB. However, they presented with higher Geriatric Depression Scale and the screening scale “Age is not a problem” scores. Patients with osteoporosis presented with lower quality of life and health status, and higher intensity of pain syndrome at the time of examination as well as 7 days prior to examination (Table 4).

**Table 4:** Results of CGA according to the presence or absence of osteoporosis in patients aged ≥ 65 years (*n* = 4269) (values in bold indicates statistical difference)

Indicator		Osteoporosis (*n* = 1101)	No osteoporosis (*n* = 3168)	*p*
Screening scale “age is not a problem.” points*		3 (2; 4)	2 (1; 4)	** < 0.001**
SPPB. points *		5 (2; 8)	6 (3; 9)	** < 0.001**
Hand grip strength. kg*	20 (15; 26)	22 (16; 30)	** < 0.001**	
	15 (10; 20)	16 (11; 22)	** < 0.001**	
Decrease in hand grip strength. %		73.7	69.8	**0.022**
Gait velocity. m/s*		0.57 (0.44; 0.82)	0.64 (0.47; 0.83)	**0.026**
Decrease in gait velocity. %		57.7	55.4	0.220
Basic activity scale in everyday life (Barthel index). points Me (25%; 75%) M ± SD		95 (80; 100) 85.3 ± 19.7	95 (90; 100) 89.7 ± 17.0	** < 0.001**
Lawton Instrumental Activities of Daily Living Scale. points Me (25%; 75%) M ± SD		7 (5; 8) 6.0 ± 2.2	7 (5; 8) 6.3 ± 2.1	** < 0.001**
MNA (Mini Nutritional Assessment) short-form (screening part). points Me (25%; 75%) M ± SD		12 (10; 13) 11.1 ± 2.2	12 (11; 13) 11.8 ± 2.1	** < 0.001**
Mini-cog test. points*		3 (2; 4)	3 (2; 4)	0.283
Geriatric depression scale points*		5 (3; 9)	4 (2; 7)	** < 0.001**
Self-assessment of the quality of life according to VAS. points*		6 (5; 8)	7 (5; 8)	** < 0.001**
Self-assessment of health status according to VAS. points*		5 (4; 7)	6 (5; 7)	** < 0.001**
Self-assessment of pain at the time of examination according to VAS. points*		4 (1; 5)	3 (0; 5)	** < 0.001**
Self-assessment of pain for the last week according to VAS. points*		5 (3; 7)	4 (1; 6)	** < 0.001**

^*^Results are presented as Me (25%; 75%)

Patients with osteoporosis used assistive products more often (with the exception of spectacles/lenses which, however, they tended to use more frequently), and their number per patient was significantly higher than in those without osteoporosis (Table 5).

**Table 5:** Frequency of use of assistive products according in relation to the occurrence of osteoporosis in patients aged ≥ 65 years (values in bold indicates statistical difference)

Indicator	Osteoporosis (*n* = 1101)	No osteoporosis (*n* = 3168)	*p*
Use of aids, %	95.7	91.5	** < 0.001**
Number of aids			** < **
Me (25%; 75%) M ± SD	2 (2; 4) 2.8 ± 1.6	2 (1; 3) 2.1 ± 1.3	**0.001**
Spectacles/lenses, %	81.1	78.8	0.097
Hearing aid, %	8.7	6.8	**0.040**
Dentures, %	65.3	57.9	** < 0.001**
Cane, %	39.3	30.4	** < 0.001**
Crutches, %	3.6	2.0	**0.002**
Walkers, %	5.6	3.4	**0.001**
Wheel-chair, %	2.9	1.5	**0.002**
Orthopedic shoes, %	10.4	3.2	** < 0.001**
Orthopedic insoles, %	19.4	6.9	** < 0.001**
Spinal brace, %	9.9	2.9	** < 0.001**
Incontinence pads, %	19.6	11.8	** < 0.001**
Diapers/underpads, %	8.9	4.8	** < 0.001**
Assistive devices to facilitate mobility (cane, crutches, walkers, wheel-chair), %	44.1	33.4	** < 0.001**
Absorbent underwear for urinary/fecal incontinence (incontinence pads, diapers, underpads), %	24.2	14.6	** < 0.001**

Patients with osteoporosis showed a higher incidence of all GSs, except for orthostatic hypotension and hearing loss (Table 6); and the most common GSs were chronic pain syndrome (95%), basic dependence in everyday life (71%), frailty syndrome (68%), cognitive impairment (65%), instrumental dependence in everyday life (58%), probable depression (57%), and urinary incontinence (57%).

**Table 6:** Incidence of geriatric syndromes in relation to osteoporosis in patients aged ≥ 65 years (values in bold indicates statistical difference)

Indicator	Osteoporosis (*n* = 1101)	No osteoporosis (*n* = 3168)	*p*
Chronic pain syndrome	95.1	84.5	** < 0.001**
Basic dependence in everyday life	70.8	57.9	** < 0.001**
Instrumental dependence in everyday life	57.9	53.0	**0.005**
Frailty syndrome	68.0	60.8	** < 0.001**
Cognitive impairments	65.0	59.5	**0.003**
Probable depression	57.2	45.0	** < 0.001**
Urinary incontinence	57.3	41.1	** < 0.001**
Falls over the previous year	45.4	25.1	** < 0.001**
Sensory deficit (any)	18.2	14.5	**0.004**
Hearing loss	13.1	11.4	0.145
Vision deficit	6.8	4.5	**0.002**
Malnutrition	10.0	4.5	** < 0.001**
Orthostatic hypotension	8.1	7.9	0.845
Fecal incontinence	7.6	3.9	** < 0.001**
Bedsores	3.5	1.9	**0.002**

A one-way regression analysis adjusted for age and gender. GSs were considered as a dependent variable, and the presence of osteoporosis, age (as an extended variable), and gender were independent variables. Results demonstrated that osteoporosis is associated with almost all GSs, with the exception of orthostatic hypotension, hearing loss, and cognitive impairment (OR from 1.19 to 3.10) (Table 7).

**Table 7:** Association between osteoporosis and geriatric syndromes in patients aged ≥ 65 years (one-way regression analysis as adjusted for age and gender) (*n* = 4269 (values in bold indicates statistical difference)

Geriatric syndromes	OR	95% CI	*p*
Instrumental dependence in everyday life	1.19	1.02–1.39	**0.027**
Sensory deficit (any)	1.22	1.00–1.48	**0.047**
Frailty syndrome	1.22	1.04–1.42	**0.013**
Vision deficit	1.44	1.06–1.94	**0.018**
Probable depression	1.52	1.32–1.75	** < 0.001**
Basic dependence in everyday life	1.53	1.31–1.80	** < 0.001**
Urinary incontinence	1.66	1.43–1.92	** < 0.001**
Fecal incontinence	1.94	1.45–2.60	** < 0.001**
Bedsores	1.97	1.29–3.01	**0.002**
Malnutrition	2.28	1.75–2.97	** < 0.001**
Falls over the previous year	2.28	1.97–2.64	** < 0.001**
Chronic pain syndrome	3.10	2.32–4.16	** < 0.001**

Dependent variable: geriatric syndromes

Multivariate regression analysis (adjusted for age and gender) included 12 GSs with a significance level of *p* < 0.05 based on the results of one-way analysis; the presence of osteoporosis was considered a dependent variable, and GSs, age (as an extended variable), and gender were considered independent variables. Multivariate analysis revealed that five of them were independently associated with osteoporosis and the likelihood of the occurrence of osteoporosis was multiplied 1.2–2.5 times, and in women, the risk of osteoporosis is 84% higher than in men (Table 8). The order of variables inclusion in the model was as follows: falls in the previous year, female gender, chronic pain syndrome, malnutrition, urinary incontinence, and probable depression.

**Table 8:** Associations between osteoporosis and geriatric syndromes in patients aged ≥ 65 years (multivariate regression analysis as adjusted for age and gender)

Predictors	OR	95% CI	*p*
Female gender	1.84	1.54–2.20	< 0.001
Probable depression	1.19	1.02–1.38	0.027
Urinary incontinence	1.37	1.18–1.59	< 0.001
Malnutrition	1.91	1.45–2.52	< 0.001
Falls over the previous year	1.94	1.67–2.25	< 0.001
Chronic pain syndrome	2.46	1.83–3.32	< 0.001

Dependent variable: osteoporosis

## Discussion

The presented subanalysis of data of the Russian epidemiological study EVKALIPT reveals that the prevalence of osteoporosis in patients based on the survey was 11.8% (*n* = 507). In addition, a group of patients with a history of low-energy fractures (*n* = 594; 15.7%) was identified according to modern criteria, and also who had osteoporosis; however, it was not diagnosed in time. For further analysis, these patients were combined into one group with patients with registered osteoporosis (osteoporosis group: *n* = 1101). Consequently, the prevalence of osteoporosis in patients aged 65 years and older increases to 25.6% with a predominance in women. Most guidelines and publications include information obtained in the early 2000s based on the results of a cluster cross-sectional study in a random sample of one of the districts of Moscow, when osteoporosis was diagnosed in patients aged 50 years and older based on changes in DXA densitometry in spine and/or proximal femur, and was detected in 33.8% of women and 26.9% of men [4]. However, we examined the indices of patients 15 years younger in this study. The prevalence of osteoporosis is known to increase with age [12]. In our study, this trend was also demonstrated and the maximum prevalence was recorded in patients over 85 years of age (29.1%) (Fig. 1). According to the social research program conducted by the US National Center for Health Statistics, National Health and Nutrition Examination Survey, from 1988 to 1994, 1.3 and 1.6% of men and 12.1 and 9.7% of women aged 75–84 years and 85 years and older, respectively, were surveyed and reported having osteoporosis; however, densitometry detected this disease already in 6.4 and 13.7% of men and 32.5 and 50.5% of women in the corresponding age categories [13]. Thus, in the Russian study EVKALIPT, we established a similar awareness (11.6%) about the presence of osteoporosis. The lack of information about instrumental examination, and in particular densitometry, limits our data obtained. In 2017–2018, in the USA, the prevalence of osteoporosis in patients over 65 years old was studied based on instrumental data alone (low BMD according to densitometry results), and it amounted to 17.7% [14]. In a European study, among patients of all ages with osteoporosis diagnosed based on a decrease in BMD in the femur, the overall prevalence was 5.6% [15].

According to Russian experts in the field of epidemiology, it has been established that the prevalence of osteoporosis in women in the Russian Federation is comparable to global data, and Russian women are of average risk of osteoporosis on a pair with residents of North America and Western Europe, and in Russian men, it exceeds the prevalence of osteoporosis in North America and Western Europe [4]. In the EVKALIPT study, women obviously predominated in the osteoporosis group among patients aged 65 years and older (Table 3). Thus, in general, the prevalence of osteoporosis in Russia is comparable to that in other countries.

The social and medical significance of osteoporosis is determined by the fractures caused by it [5]. In the EVKALIPT study, 17% of patients had a history of low-energy fractures (Table 2). According to localization, fractures of the radial bone in the history prevailed (10.8%), proximal femur fractures were less common (6.8%), and fractures of the vertebrae due to a fall from standing height in the history rarely occurred (3.7%) (Table 2). Fractures of the radial bone were more common in patients aged 75–84 years, and fractures of the proximal femur and vertebrae were registered more often in patients older than 85 years. At the age of 50 years, the probability of fracture of the proximal femur during the subsequent life in the Russian Federation is 4% for men and 7% for women. In 2010, the number of fracture cases of the proximal femur in Russia was 112,000 cases; by 2035, due to increased life expectancy alone, it will increase by 36% in men and 43% in women, and will amount to 159 thousand cases per year [16]. According to available statistics, the prevalence of bone fractures in Russia is 18.6 cases per 1000 adult population, or 21.5% [4], which is somewhat higher than in our study. Osteoporotic vertebral fractures represent a serious problem, which is due not only to their high real prevalence and low detectability, but also to a significant impact on the decrease in the quality of life [17]. Studies conducted earlier in three cities of Russia (Moscow, Yekaterinburg, and Yaroslavl) showed that the prevalence of these fractures is 7.2% and higher in men and 7% and higher in women, but again at a younger age [18]. Nevertheless, the results of detection of vertebral fractures in the EVKALIPT study (3.7%) still indicate their insufficient diagnostics in patients over 65 years of age. At the same time, the vast majority of them had fractures of the radial bone (Table 2). This type of fracture (distal part of the forearm) is one of the most common fractures in a fall from standing height. According to an epidemiological study in Russia, its frequency was 426/100,000 of the population, exceeding the incidence of femoral fracture by 3–7 times in men and 4–8 times in women, and significantly prevailing in women. In our study, the first fracture of the radial bone most often occurred at the age of 65–74 years, but a repeated fracture of the same location was registered already at the age of 75–84 years, which is consistent with the data that the risk of a subsequent fracture after the initial fracture increases by 1.6–4.3 times at any age [19].

However, it is noteworthy that in 594 (15.77%) patients with a history of fracture in the subanalysis presented, osteoporosis was not diagnosed and, accordingly, no treatment was prescribed. When analyzing data from 6 European countries (France, Spain, Italy, Great Britain, Sweden), 60–85% of women with a history of fracture also did not receive subsequent treatment for osteoporosis [20]. Our study revealed that only 12 (4.7%) patients received antiresorptive therapy, 1 (0.4%) patient received bone anabolic therapy, and 9 (3.5%) patients received both antiresorptive and bone-metabolic therapy; however, 91.4% of patients still confirmed the prescription of vitamin D and calcium supplements. These data indicate insufficiently complete treatment of osteoporosis. Similar results were obtained in Europe; thus, 71% of women with indications for anti-osteoporotic treatment did not receive this therapy [15]. These findings can be due to a number of factors, including the low awareness of doctors and patients about the need for osteoporosis treatment, the need for long-term treatment of osteoporosis, the high cost of drugs for the treatment of osteoporosis, and the large number of drugs taken for other diseases. Thus, in the osteoporosis group, a greater number of patients were recorded who took 5 or more drugs. Polypharmacy is a GS which is often cited as a potential barrier to adherence to the regimen of drug intake, used to treat osteoporosis, and is an independent negative factor in long-term therapy [21]. It has been revealed that women taking medications for other diseases were less adherent to osteoporosis therapy, and those who used more than ten drugs had a 1.87-fold risk of being non-adherent compared to those who did not use medications [22].

Patients with osteoporosis have a high comorbidity, a burdened geriatric status [12]. Thus, in our study, the greatest number of GSs was revealed in patients with osteoporosis, with the exception of orthostatic hypotension and hearing loss (Table 6). In addition, osteoporosis, being associated with almost all GSs (OR 1.19–3.10), was not associated with orthostatic hypotension, hearing loss, and cognitive impairment (Table 7). This could be explained by the fact that patients over 65 years of age with osteoporosis frequently used hearing aids than patients without osteoporosis (Table 5). Osteoporosis, as a change in bone mineral density, obviously develops at an earlier age than the main many GSs. However, osteoporosis is diagnosed at an older age, when GSs already appear and are detected (addiction in everyday life, various sensitivity disorders, frailty syndrome, malnutrition, signs of immobility, such as fecal incontinence, bedsores, and falls, as well as chronic pain). It should be noted that in patients of the age categories considered, significant hormonal changes occur in the body, namely the levels of growth hormone, sex hormones, and insulin-like growth factor as well as their bioavailability (decreases). Such changes lead to impaired osteogenesis and increased bone resorption. A significant contribution to the pathogenesis of bone aging is due to vitamin D deficiency that increases with age and is aggravated by such GSs as malnutrition. Such an association of GSs and osteoporosis arises possibly, among other things, due to increased immune inflammation and secretion of pro-inflammatory interleukins (IL-1, IL-6) and TNF. Aged patients have decreased glomerular filtration rate which exacerbates age-related vitamin D deficiency. All these changes initiate the progression of osteoporosis in patients over 65 years of age [23, 24]. Thus, geriatric syndromes are associated with the presence of osteoporosis and its severity. The results of the association of most GSs and osteoporosis are consistent with the findings obtained in a study conducted at the Russian Gerontological Research and Clinical Center among patients of the geriatric therapy department, which revealed that patients with osteoporosis had frailty syndrome, malnutrition, and physical inactivity significantly more often (*p* < 0.001) [25]. So the geriatric status of older patients with osteoporosis tends to be worse.

It should be noted that weight loss and a decrease in muscle mass/strength are associated with frailty syndrome and are signs of sarcopenia [26]. Sarcopenia is a progressive generalized skeletal muscle disorder associated with a high risk of adverse outcomes, including falls, fractures, and physical disability as well as death. Sarcopenia and osteopenia have common risk factors and pathogenesis and are associated with myogenesis and osteogenesis. The combination of sarcopenia and osteoporosis/osteopenia is called sarcoosteopenia. In 2009, this term was first used in elderly patients with a higher risk of falls, fractures, disability, and decrepitude [27]. In our study, muscle strength was assessed using carpal dynamometry (Table 4). In the osteoporosis group, muscle strength was reduced in 73% of patients; in the same group, the SPPB score was lower (Table 4). Thus, the decrease in these indicators in the osteoporosis group was due to osteosarcopenia. Age-related changes in the hormonal and metabolic status in combination with the majority of GSs and the probable development of osteosarcopenia, as well as changes in the life status of a person with age, could explain the results of the multivariate analysis (Table 8).

## Conclusion

Osteoporosis is a severe age-related disease that can lead to poor outcomes. Geriatric syndromes are associated with the presence of osteoporosis and its severity; so the geriatric status of older patients with osteoporosis tends to be worse. The association of osteoporosis with a large number of GSs aggravates the condition of these patients making treatment difficult. Therefore, scheduled, early, and timely diagnosis of osteoporosis (careful assessment of the history of falls, fractures, and their localization, as well as changes in anthropometric parameters, namely reduced height) and GSs, as well as development of an individual treatment plan to alleviate the condition of these patients, is of great clinical utility. “Treat the patient the way you would like to be treated” is an ancient truth which is at the core of medicine. It is an obvious fact that important, new, and interesting conclusions were made in the EVKALIPT study, despite its complicated, time-consuming, and diverse nature. Success and efficiency in the management are achieved only in a team approach to solving problems. Thus, it is necessary to adopt additional programs at the state level to introduce information about GSs and opportunities to work with elderly patients; timely (early) detection and correction of osteoporosis; and dissemination of information among medical workers, patients, and their relatives.

### Limitations of the study

1. One of the disadvantages is the fact that the diagnosis of osteoporosis was revealed on the past medical history, which limits us in comparison with other studies where DXA was performed.
2. The study focuses on 3 localizations of osteoporotic fractures, which we believe are the most important and relevant. Other fractures are less often taken into account by patients and doctors, and are more difficult to statistically analyze.

## Data Availability

The data that support the findings of this study are available on request from the corresponding author [OnuchinaY.S.]. The data are not publicly available due to them containing information that could compromise research participant privacy/consent.
