# Correction: The association of osteoporosis and geriatric syndromes in the elderly: data from the Russian epidemiological study EVKALIPT

**DOI:** 10.1007/s11657-023-01228-8

**Published:** 2023-03-02

**Authors:** Ekaterina N. Dudinskaya, Natalia M. Vorobyeva, Julia S. Onuchina, Lubov V. Machekhina, Elena V. Selezneva, Lilia N. Ovcharova, Yulia V. Kotovskaya, Olga N. Tkacheva

**Affiliations:** 1 Age-Related Endocrine and Metabolic Disorders Laboratory, Russian Gerontology Research and Clinical Centre, Pirogov Russian National Research Medical University, Moscow, Russia; 2 Laboratory of Cardiovascular Aging, Russian Gerontology Research and Clinical Centre, Pirogov Russian National Research Medical University, Moscow, Russia; 3 Department of Aging Diseases, Faculty of Additional Professional Education, Russian Gerontology Research and Clinical Centre, Pirogov Russian National Research Medical University, Moscow, Russia; 4 Institute for Social Policy, National Research University “Higher School of Economics”, Moscow, Russia; 5 Russian Gerontology Research and Clinical Centre, Pirogov Russian National Research Medical University, Moscow, Russia; 6 Russian Academy of Sciences, Moscow, Russia; 7 Biomarkers of aging Laboratory, Russian Gerontology Research and Clinical Centre, Pirogov National Research Medical University, Moscow, Russia

**Correction: Archives of Osteoporosis (2023) 18:30**

10.1007/s11657-023-01217-x

The article “The association of osteoporosis and geriatric syndromes in the elderly: data from the Russian epidemiological study EVKALIPT”, written by Ekaterina N. Dudinskaya, Natalia M. Vorobyeva, Julia S. Onuchina, Lubov V. Machekhina, Elena V. Selezneva, Lilia N. Ovcharova, Yulia V. Kotovskaya & Olga N. Tkacheva, was originally published electronically on the publisher’s internet portal on 13 February 2023 without open access. With the author(s)’ decision to opt for Open Choice the copyright of the article changed on 14 February 2023 to © The Authors 2023 and the article is forthwith distributed under a Creative Commons Attribution 4.0 International License, which permits use, sharing, adaptation, distribution and reproduction in any medium or format, as long as you give appropriate credit to the original author(s) and the source, provide a link to the Creative Commons licence, and indicate if changes were made. The images or other third party material in this article are included in the article’s Creative Commons licence, unless indicated otherwise in a credit line to the material. If material is not included in the article’s Creative Commons licence and your intended use is not permitted by statutory regulation or exceeds the permitted use, you will need to obtain permission directly from the copyright holder. To view a copy of this licence, visit http://creativecommons.org/licenses/by/4.0.

